# Tumour necrosis is a postoperative prognostic marker for pancreatic cancer patients with a high interobserver reproducibility in histological evaluation

**DOI:** 10.1038/sj.bjc.6605854

**Published:** 2010-08-24

**Authors:** N Hiraoka, Y Ino, S Sekine, H Tsuda, K Shimada, T Kosuge, J Zavada, M Yoshida, K Yamada, T Koyama, Y Kanai

**Affiliations:** 1 Pathology Division, National Cancer Center Research Institute, 5-1-1 Tsukiji, Chuo-ku, Tokyo 104-0045, Japan; 2 Clinical Laboratory Division, National Cancer Center Hospital, 5-1-1 Tsukiji, Chuo-ku, Tokyo 104-0045, Japan; 3 Hepato-Biliary and Pancreatic Surgery Division, National Cancer Center Hospital, 5-1-1 Tsukiji, Chuo-ku, Tokyo 104-0045, Japan; 4 Institute of Organic Chemistry and Biochemistry, Flemingovo nam.2, 166 10 Prague 6, Czech Republic

**Keywords:** necrosis, pancreatic cancer, prognostic factor, interobserver reproducibility, hypoxia

## Abstract

**Background:**

Tumour necrosis reflects the presence of hypoxia, which can be indicative of an aggressive tumour phenotype. The aim of this study was to investigate whether histological necrosis is a useful predictor of outcome in patients with pancreatic ductal carcinoma (PDC).

**Methods:**

We reviewed histopathological findings in 348 cases of PDC in comparison with clinicopathological information. We counted small necrotic foci (micronecrosis) as necrosis, in addition to massive necrosis that had been only defined as necrosis in previous studies. The reproducibility of identifying histological parameters was tested by asking five independent observers to blindly review 51 examples of PDC.

**Results:**

Both micronecrosis and massive necrosis corresponded to hypoxic foci expressing carbonic anhydrase IX detected by immunohistochemistry. Multivariate survival analysis showed that histological necrosis was an independent predictor of poor outcome in terms of both disease-free survival (DFS) and disease-specific survival (DSS) of PDC patients. In addition, metastatic status, and lymphatic, venous, and intrapancreatic neural invasion were independent prognostic factors for shorter DFS and metastatic status, margin status, lymphatic invasion, and intrapancreatic neural invasion were independent prognostic factors for DSS. The interobserver reproducibility of necrosis identification among the five independent observers was ‘almost perfect’ (*κ*-value of 0.87).

**Conclusion:**

Histological necrosis is a simple, accurate, and reproducible predictor of postoperative outcome in PDC patients.

Pancreatic cancer (pancreatic ductal carcinoma (PDC)) is the fourth and fifth leading cause of cancer-related death in the United States and Japan, respectively (Center for Cancer Control and Information Services and National Cancer Center, 2009; Jemal *et al*, 2009). Because of its aggressive growth and early metastatic dissemination, the overall 5-year survival rate for patients with pancreatic cancer is 3–5%, and that of patients treated by curative resection is 15–25% (Klöppel *et al*, 2000; Lim *et al*, 2003; Hruban *et al*, 2007; Jemal *et al*, 2009). The mortality rate has not shown any obvious improvement for decades. The development of predictive biomarkers to assist the selection of patient subsets is useful for studies aimed at reducing the mortality of PDC patients, especially in phase clinical studies evaluating various therapeutic approaches (Philip *et al*, 2009).

Several histopathological parameters such as tumour size (Yeo *et al*, 1995; Sohn *et al*, 2000; Lim *et al*, 2003; Shimada *et al*, 2006), tumour histological grade (Luttges *et al*, 2000; Adsay *et al*, 2005), lymph nodal metastasis (Trede *et al*, 1990; Lim *et al*, 2003; Schnelldorfer *et al*, 2008), and lymphatic, venous (Luttges *et al*, 2000; Takai *et al*, 2003), and neural invasion (Mitsunaga *et al*, 2005, 2007) have been proposed as hallmarks predictive of postoperative outcome in patients with PDC. Stage is the most important prognosticator (Klöppel *et al*, 2000; Hruban *et al*, 2007), although majority of resectable cases were classified into advanced stage, stage IIB of the International Union Against Cancer (UICC) tumour–node–metastasis (TNM) classification and were not able to be stratified more precisely. Histopathological evaluation of PDC can be performed using routine diagnostic techniques in pathology departments without any additional equipment or special expertise. The predictive values of the above mentioned parameters are sometimes controversial and complex, and some have problems related to interobserver reproducibility. To avoid these pitfalls and to stratify PDCs in a distinct and objective manner, molecular or genetic markers have been developed, although these often require special equipment or expertise and cannot usually be performed on a routine basis. Therefore, simpler, more reproducible, and easily assessable histopathological predictors are needed.

Hypoxia is a common feature of human cancers, which induces a transcription programme mediated mainly by hypoxia-inducible factor-1*α* (HIF-1*α*) that promotes aggressive tumour phenotype (Harris, 2002; Vaupel and Mayer, 2007; Bristow and Hill, 2008). It is a prognostic indicator in many solid tumours (Vaupel and Mayer, 2007), and is often detected by examining the expression of carbonic anhydrase IX (CAIX) (Chia *et al*, 2001), which is a regulator of cellular pH and its expression is induced by HIF-1*α* (Harris, 2002; Bristow and Hill, 2008). Intratumoural hypoxia is reflected histologically by the presence of necrosis, which has also been reported to be a prognostic factor in patients with breast (Gilchrist *et al*, 1993) and bladder (Ord *et al*, 2007) cancers. Hypoxia is evident in PDCs, in which expression of HIF-1*α* and CAIX has been detected in 60–70% and 78% of cases, respectively (Kitada *et al*, 2003; Couvelard *et al*, 2005; Sun *et al*, 2007), whereas histological necrosis has been found in only 30–40% of PDCs in previous studies (Couvelard *et al*, 2005; Mitsunaga *et al*, 2005). Neither large-scale study nor multivariate survival study has been performed to evaluate prognostic value of hypoxia in PDC patients.

In this study, we found that necrotic areas were present in more than 60% of PDCs when small necrotic foci were taken into account. We also found that CAIX were expressed in and around these necrotic lesions, indicating that these areas were in a condition of hypoxia. Therefore, detection of necrosis using our definition may offer a chance of stratifying PDC patients for tissue hypoxia more accurately. In this study, with the aim of investigating whether histologically evident necrosis is useful for prediction of patient outcome among various histopathological parameters, and we reviewed histopathological findings in 348 cases of PDC in comparison with clinicopathological information.

## Materials and methods

### Study population

This study was approved by the Ethics Committee of the National Cancer Center, Japan. Clinical and pathological data were obtained through a detailed retrospective review of the medical records of all 348 patients with ductal carcinoma of the pancreas that had undergone initial surgical resection between 1990 and 2005 at the National Cancer Center Hospital. None of the patients had received any previous therapy. All patients received standard therapy appropriate for their clinical stages. The operative procedures included 228 pancreatoduodenectomies or pylorus-preserving pancreatoduodenectomies, 104 distal pancreatectomies, and 16 total pancreatectomies. Along with tumour extension, lymphadenectomy was performed at the hepatoduodenal ligament and around the abdominal aorta. All the patients included in this study underwent macroscopic curative resection, which was defined as the macroscopic removal of all gross tumours without liver metastases, macroscopic peritoneal dissemination, bulky lymph node involvement, or apparent tumour invasion around the common hepatic or superior mesenteric arteries after routine examination using intraoperative ultrasonography. All of the cases were conventional ductal carcinomas and adenocarcinomas originating in intraductal papillary mucinous neoplasms or mucinous cystic neoplasms were excluded. Secondary tumours and postneoadjuvant cases were also excluded. The clinicopathological characters of the patients were summarized in Table 1. All M1 patients had nodal metastasis around the abdominal aorta, without any other form of metastasis. Males accounted for 206 patients and females for 142; the mean patient age was 62.9 years (range, 27–87 years). Every patient was followed up in the outpatient clinic every 1–3 month during the first postoperative year, and every 6–12 months thereafter. Patients underwent physical examination, laboratory tests, chest radiography, abdominal computed tomography, and/or ultrasonography, unless there was a confirmed relapse. The tumour markers carcinoembryonic antigen and carbohydrate antigen 19-9 were also measured until relapse. Recurrence was suspected when a new local or distant metastatic lesion was found on serial images and an increase in tumour marker levels was recognised. When progression of the disease was confirmed by repeated imaging studies, the date of the first suspicious radiologic finding was used as the date of initial disease recurrence. The median follow-up period after surgery was 17.9 (1.3–210) months for the patients overall: 69 patients (19.8%) were alive at the census date (June 2009), 239 (68.7%) died because of pancreatic cancer, and 40 (11.5%) died of other causes. Postresection adjuvant therapy information was available for 327 patients, of whom 19 received chemotherapy and radiotherapy, 134 received chemotherapy only, 2 received radiotherapy only, and 172 did not receive any additional therapy.

### Pathological examination

All of the ductal carcinomas were pathologically reexamined and were classified according to the World Health Organisation classification (Klöppel *et al*, 2000), UICC TNM classification (Wittekind *et al*, 2005), and the Classification of Pancreatic Carcinoma of the Japan Pancreas Society (JPS) (Japan Pancreas Society, 2003). Surgically resected specimens were fixed in 10% formalin and cut into serial 5-mm-thick slices, horizontally in the pancreas head, and sagittally in the pancreas body and tail. All the sections were stained with hematoxylin and eosin for pathological examination. The following histopathological variables were evaluated according to the classification of JPS: tumour histological grade, nerve plexus invasion, and lymphatic, venous, and intrapancreatic neural invasion.

**Figure 1:**
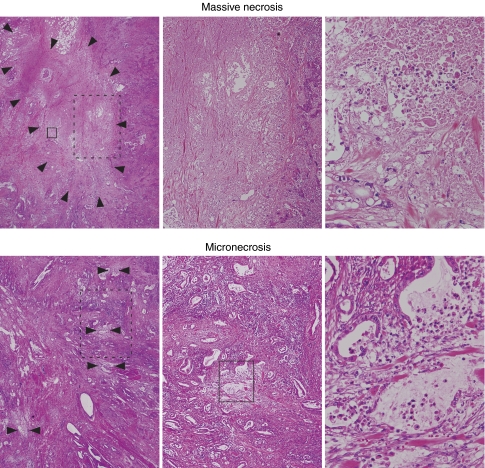
Representative histology of massive necrosis (upper columns) and micronecrosis (lower columns). Arrows indicate necrotic area. Left, centre, and right columns are in low ( × 6.25), middle ( × 20), and high magnification ( × 100), respectively. High power view of histology in right columns corresponds to the rectangle (solid line) in left or middle column. Middle power view of histology in centre columns corresponds to the rectangle (dotted line) in left columns.

Tumour necrosis in PDCs was reported previously (Couvelard *et al*, 2005; Mitsunaga *et al*, 2005), having been defined as ‘confluent cell death in invasive areas of primary cancer, visible at an objective lens magnification of × 4′ (Mitsunaga *et al*, 2005), which is the same to the definition having been mentioned in breast cancer. Tumour histology often varies among the organs in which tumours develop. We noticed that small areas of necrosis were evident in PDCs, wherein gland formation by cancer cells was ruptured, usually in association with neutrophil infiltration. We refer to this hereafter as micronecrosis, and to the former as massive necrosis. As sometimes it is difficult to differentiate necrotic lesions into either of these two patterns, we combined these two types of lesions solely as ‘necrosis’. The definition of histological necrosis is as follows. Necrosis occurs in cancer tissue regardless of its extent, and is usually found in both cancer cells and cancer stroma (Figure 1). When coagulation necrosis is extensively developed (massive necrosis), it corresponds to what was referred to as necrosis previously (Couvelard *et al*, 2005; Mitsunaga *et al*, 2005). Smaller areas of necrosis (micronecrosis) often recognised adjacent to ruptured cancer-forming tubules is almost always accompanied by neutrophil infiltration (Figure 1).

### Interobserver reproducibility in identifying histological characteristics

To test the reproducibility of identification of histological characteristics, five independent observers (SS, HT, MY, KY, and TK) were asked to review 51 examples of IDC that were consecutive cases surgically resected between 1997 and 2000. One slide was selected from each of the 51 cases. This slide was one of the slides containing the maximum cut section of the tumour. The complete set of slides from the 51 cases was sent to the five independent observers, all of whom are general surgical pathologists with no specific expertise in the pancreas, and who encounter pancreas specimens rarely. One observer (HT) specialises in breast and female genitourinary pathology and one (SS) in gastrointestinal tract pathology. These observers were provided with the definition of ‘histological necrosis’ according to Figure 1 and with the definitions for lymphatic, venous, and neural invasion stated in the classification of JPS (Japan Pancreas Society, 2003). They were asked to assess the presence of histological necrosis and each of the grades of lymphatic, venous, and neural invasion evident within each of the provided slides. These observers were blind to the identity of the original reviewers or those of each other. They were also not provided with any clinical information on the outcome of the patients.

### Immunohistochemical analysis

Immunohistochemistry was performed on formalin-fixed, paraffin-embedded tissue sections as described previously (Takahashi *et al*, 2007), using antibodies against CAIX (M75, 1 : 200) (Pastorekova *et al*, 1992; Zavada *et al*, 2000) and HIF-1*α* (54, 1 : 500, BD Transduction Laboratories, Franklin Lakes, NJ, USA). Avidin–biotin complex method and CSA system (DAKO, Glostrup, Denmark) were used for these immunohistochemistry, respectively. The sections were autoclaved in the buffer (pH 9.0, Nichirei Biosciences, Tokyo, Japan) for antigen retrieval. For immunohistochemical examination of CAIX in PDCs, we used sections of representative blocks from 203 cases of PDC. Carbonic anhydrase IX is expressed always in the crypt enterocytes of the duodenum and sometimes in normal epithelial cells of the pancreatic duct and pancreatic intraepithelial neoplasm. These cells were used as the positive control for CAIX immunohistochemistry. Immunohistochemistry was performed without primary antibody for negative control. When more than 20% of cancer cells in the specimen expressed CAIX, the case was judged as positive for CAIX in cancer cells. When there were any stromal cells expressing CAIX in cancer tissue, the case was judged as presence of stromal cells expressing CAIX.

### Statistical analysis

Comparisons of qualitative variables were performed using the *χ*^2^ test or Fisher's exact test. One-way analysis of variance was used to compare the means of three or more groups. The postoperative disease-free survival (DFS) and disease-specific survival (DSS) rates were calculated by the Kaplan–Meier method. Univariate analysis was performed for prognostic factors using the log-rank test. The factors found to be predictive by univariate analysis were subjected to multivariate analysis using the Cox proportional hazards model (backward elimination method). Interobserver agreement (reproducibility) was tested by obtaining the κ-scores (Fleiss, 1971; Landis and Koch, 1977). Differences at *P*<0.05 were considered statistically significant. Statistical analyses were performed with StatView-J 5.0 software (Abacus Concepts, Berkeley, CA, USA).

## Results

### Correspondence of both massive necrosis and micronecrosis with hypoxic foci

**Figure 2:**
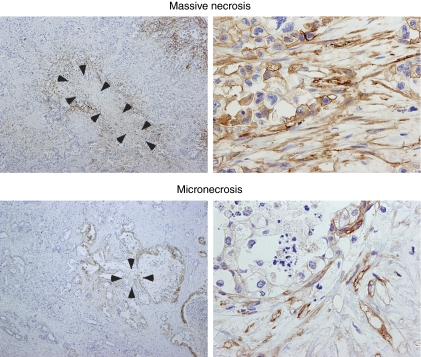
Hypoxia is reflected by the presence of massive necrosis or micronecrosis. Expression of CAIX is immunohistochemically detectable in both cancer cells and stromal cells within or around areas of massive necrosis (upper columns) and micronecrosis (lower columns). Carbonic anhydrase IX is expressed in plasma membrane. Arrows indicate necrotic area. Low power view (left columns) and high power view (right columns).

Immunohistochemical analysis revealed that CAIX was expressed in cancer cells or stromal cells within or around areas of both massive necrosis and micronecrosis (Figure 2).

### Prognostic significance of the presence of hypoxic foci detected by expression of CAIX in cancer stromal cells

**Figure 3:**
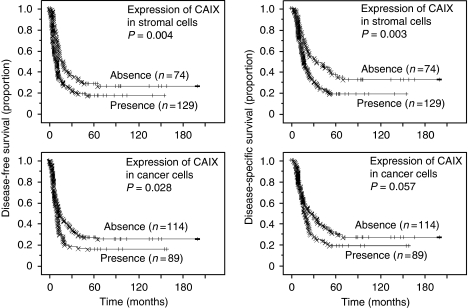
Kaplan–Meier survival curves showing the comparison of disease-free survival between high and low expression of CAIX (*P-*values obtained from log-rank test) (left columns). Kaplan–Meier survival curves showing the comparison of disease-specific survival between high and low expression of CAIX (*P*-values obtained from log-rank test) (right columns).

Survival analysis showed that the presence of hypoxic foci with expression of CAIX in stromal cells in cancer tissue was closely associated with shorter DFS (*P*=0.004) and DSS (*P*=0.003) (Figure 3). The presence of cancer cells expressing CAIX was also associated with shorter survival rates (Figure 3), although its occasional expression in cancer cells forming well-differentiated glands distant from necrotic areas probably indicated a cellular phenotype similar to that of normal ductal epithelial cells, and was unrelated to hypoxia. Then we used expression of CAIX in cancer stromal cells as hypoxic marker in this study. Multivariate Cox regression analysis revealed that the presence of hypoxic foci was an independent predictor of shorter DFS (*P*=0.005) and DSS (*P*=0.011) (Supplementary Table 1). The presence of necrosis was significantly correlated with the presence of hypoxic foci (Table 1). More CAIX-expressing cells were found in larger areas of necrosis.

### Histopathological evaluation of PDC

Table 1 lists the clinicopathological features of patients with PDC. When correlations with these clinicopathological features were analyzed, the presence of necrosis was found to be more likely in cases with large tumours (*P*=0.004), higher tumour status (*P*=0.023), presence of nodal metastasis (*P*=0.030), presence of distant metastasis (*P*=0.049), higher TNM stage (*P*=0.009), poorer tumour differentiation (*P*<0.0001), and more frequent venous invasion (*P*=0.0002).

### Prognostic significance of the histopathological valuables

**Figure 4:**
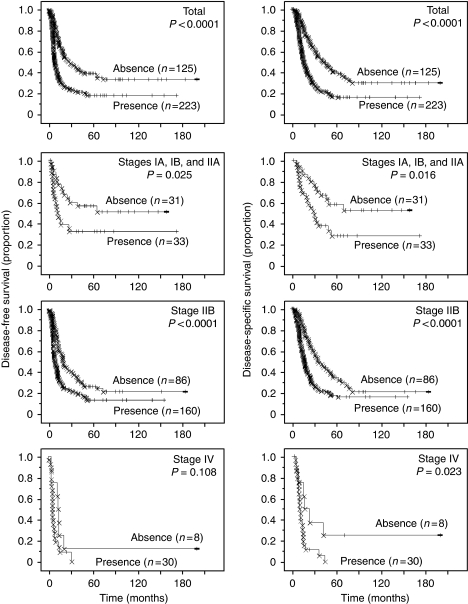
Kaplan–Meier survival curves showing a comparison of disease-free survival between cases in which histological necrosis was present and absent (*P*-values obtained by log-rank test) (left columns). Kaplan–Meier survival curves showing a comparison of disease-specific survival between cases, in which histological necrosis was present and absent (*P*-values obtained by log-rank test) (right columns).

Survival analysis demonstrated an association between the presence of necrosis and shorter DFS (*P*<0.0001; HR=2.007; 95% CI: 1.531–2.630) and DSS (*P*<0.0001; HR=2.196; 95% CI: 1.659–2.905) (Figure 4). A similar association was found in patients with PDC at each TNM stages (Figure 4).

The average survival periods for patients having PDC with and without necrosis were 24.62±1.42 months and 47.36±2.75 months, respectively. One-year survival rates for patients having PDC with and without necrosis were 63.3±3.3% and 89.1±2.9%, respectively; the 2-year rates were 36.2±3.4% and 69.0±4.3%, and the 5-year rates were 17.1±2.9% and 40.1±4.8%.

Multivariate Cox regression analysis showed that necrosis (*P*<0.0001; HR=1.853; 95% CI: 1.407–2.440), metastatic status, lymphatic invasion, venous invasion, and intrapancreatic neural invasion were independent predictors of DFS, and that necrosis (*P*<0.0001; HR=2.238; 95% CI: 1.686–2.971), metastatic status, margin status, lymphatic invasion, and intrapancreatic neural invasion were independent predictors of DSS (Table 2).

When massive necrosis and micronecrosis were separated as distinct variables, univariate survival analysis demonstrated an association between the presence of massive necrosis and shorter DFS (*P*<0.0001) and DSS (*P*<0.0001), and between the presence of micronecrosis and shorter DFS (*P*=0.004) and DSS (*P*=0.001) (Supplementary Table 2). Multivariate Cox regression analysis showed that massive necrosis (*P*=0.0006) and micronecrosis (*P*=0.0004) were independent predictors of shorter DFS and that massive necrosis (*P*<0.0001) and micronecrosis (*P*<0.0001) were independent predictors of shorter DSS (Supplementary Table 2).

### Reproducibility of necrosis identification by independent observers

Interobserver agreement (reproducibility) regarding the identification of necrosis among the five independent observers who reviewed the 51 slides blindly had a *κ*-value of 0.87. On the other hand, the corresponding κ-values for grading (0–3) of lymphatic, venous, and neural invasion were 0.11, 0.11, and 0.31, respectively. According to the widely used statistical chart that grades the strength of agreement (Fleiss, 1971; Landis and Koch, 1977) into 6 categories (poor (*κ*-value, <0.00), slight (0.00–0.20), fair (0.21–0.40), moderate (0.41–0.60), substantial (0.61–0.80), and almost perfect (0.81–1.00)), the agreement for identification of necrosis and the grading for lymphatic, venous, and neural invasion were categorized as ‘almost perfect’, ‘slight’, ‘slight’, and ‘fair’, respectively. When the grades for lymphatic, venous, and neural invasion were combined into two categories (grades 0 and 1 and grades 2 and 3) as used in the survival analysis, the *κ*-values for lymphatic, venous, and neural invasion were 0.55 (moderate), 0.62 (substantial), and 0.62 (substantial), respectively.

Survival analysis was performed using the data for necrosis identification by the five independent observers, and this yielded similar results, that is, patients having PDC with necrosis showed significantly shorter survival. *P*-values calculated for each of these analyses were 0.0004, 0.0005, 0.002, 0.005, 0.006, and 0.008 for DFS, and 0.0001, 0.0004, 0.003, 0.005, 0.005, and 0.008 for DSS.

## Discussion

Pancreatic ductal carcinoma is one of the most aggressive cancers, with almost equivalent incidence and mortality rates. Several histopathological prognostic variables for patients with PDC have been reported (Trede *et al*, 1990; Yeo *et al*, 1995; Luttges *et al*, 2000; Sohn *et al*, 2000; Lim *et al*, 2003; Takai *et al*, 2003; Adsay *et al*, 2005; Mitsunaga *et al*, 2005, 2007; Shimada *et al*, 2006; Schnelldorfer *et al*, 2008). These are sometimes controversial and complex, and some have problems related to interobserver reproducibility. We need prognostic indicators being simpler, more reproducible, and accurate.

In this study, we reviewed the histopathological findings in 348 cases of PDC in comparison with the corresponding clinicopathological information, and obtained several histopathological prognosticators of both DFS and DSS for patients with PDC by univariate survival analyses employing histological necrosis, tumour size, tumour status, node status, metastatic status, margin status, nerve plexus invasion, and lymphatic, venous, and intrapancreatic neural invasion as variables. Multivariate survival analyses revealed that histological necrosis was an independent predictive factor for both shorter DFS (*P*<0.0001) and shorter DSS (*P*<0.0001) of PDC patients (Table 2). In addition, metastatic status, lymphatic invasion, venous invasion, and intrapancreatic neural invasion were factors that were independently predictive of shorter DFS, whereas metastatic status, margin status, lymphatic invasion, and intrapancreatic neural invasion were factors independently predictive of shorter DSS (Table 2). Necrosis was also able to predict patient outcome in populations at stage IIB, into which the majority of resectable PDCs were stratified (Figure 4). Furthermore, the reproducibility of necrosis identification was found to be ‘almost perfect’ (*κ*-value, 0.87) when the 51 slides of PDC were assessed by five independent observers. In contrast, the reproducibility of the systems of grading for lymphatic, venous, and neural invasion was low with low *κ*-values. Moreover, these five independent observers all provided the same result, that is, that patients having PDC with necrosis showed significantly shorter survival in terms of both DFS and DSS. These findings indicate that histological necrosis is a simple, accurate, and reproducible predictor of postoperative outcome for PDC patients.

Histological necrosis was found in 223 (64.1%) out of 348 cases of PDC. We defined necrosis as covering both massive necrosis and micronecrosis, the latter being often evident in PDCs, although not noted previously or being hidden as smaller foci (Couvelard *et al*, 2005; Mitsunaga *et al*, 2005). The expression of CAIX (Figure 2) and HIF-1*α* (data now shown) in and around the necrotic areas was detected immunohistochemically, together with that the presence of necrosis was significantly correlated with expression of CAIX (Table 1), indicating that both patterns of necrosis were closely related to hypoxia. Hasebe's group reported that necrosis was an independent prognostic factor in both DFS and DSS when they investigated histopathological findings in 101 PDC patients (Nakatsura *et al*, 1997; Mitsunaga *et al*, 2005). Couvelard *et al* (2005) reported that necrosis was associated with poorer DSS in univariate analysis, and also showed that necrosis was significantly associated with CAIX expression. Only 30–40% of PDC cases had necrosis in those studies, whereas 60–80% of PDCs had hypoxia, as necrosis was defined roughly by these two groups as massive necrosis only.

When massive necrosis and micronecrosis were separated as distinct variables, univariate and multivariate survival analyses demonstrated that both types of necrosis were significantly associated with shorter DFS and DSS (Supplementary Table 2). Massive necrosis and micronecrosis was detected in 27.9 and 43.4% (overlapped 7.2%) of PDCs in our series, respectively. These findings suggest that the presence of necrosis, even when the lesion is small, is closely associated with patient outcome, probably because the presence of necrosis represents a hypoxia-associated aggressive tumour phenotype. In fact, in this series, the presence of necrosis was significantly correlated with a large tumour size, higher T factor, presence of nodal and distant metastasis, higher TNM stage, more severe venous invasion, and a higher tumour histological grade (Table 1), suggesting that necrosis is positively correlated with tumour growth, invasion, and angiogenesis, that is, an aggressive phenotype.

Hypoxia is a characteristic of invasive cancers that can lead to the development of an aggressive phenotype through a mechanism mediated mainly by HIF-1*α*, which includes cell immortalisation and dedifferentiation, pH regulation, autocrine growth/survival, angiogenesis, invasion/metastasis, and resistance to chemotherapy (Semenza, 2006, 2009; Grothey and Galanis, 2009). In fact, the presence of hypoxic foci with expression of CAIX was an independent worse prognostic factor for PDC patients (Supplementary Table 1) that we demonstrated at first time using large series of cases. Recently, HIF-1 targeting therapy and anti-angiogenesis therapy have been reported to yield promising anti-cancer effects (Sessa *et al*, 2008; Grothey and Galanis, 2009; Semenza, 2009). It is suggested that evaluation of histological necrosis would be useful not only for decision making about postoperative clinical management, but also for stratifying patients for clinical trials aimed at evaluating HIF-1 targeting or anti-angiogenesis therapies.

In conclusion, we reviewed the histopathological findings in 348 PDCs for which the presence of necrosis was redefined, and found that histological necrosis was an independent predictor of shorter DFS and DSS for the affected patients. Interobserver reproducibility for the detection of necrosis was assessed as ‘almost perfect’ when 51 slides of PDC were reviewed by five independent observers. These findings indicate that histological necrosis is a simple, accurate, and reproducible predictor of postoperative outcome in PDC patients.

## Figures and Tables

**Table 1:** Relationship between clinicopathological characteristics and histological necrosis

		**Necrosis**		
**Characteristics**	**No. of patients**	**Presence**	**Absence**	***P***
*Age, years*				
<60	123	75	48	
⩾60	225	148	77	0.414
				
*Sex*				
Male	206	143	63	
Female	142	80	62	**0.017**
				
*Localisation*				
Pancreas head	228	147	81	
Pancreas body or tail	109	70	39	1.000
				
*Size (mm)*				
<30	83	42	41	
⩾30	265	181	84	**0.004**
				
*Pathologic tumour status*				
T1	6	1	5	
T2	3	3	0	
T3	339	219	120	
T4	0	0	0	**0.023** a
				
*Pathologic node status*				
N0	64	33	31	
N1	284	190	94	**0.030**
				
*Pathologic metastasis status*				
M0	310	193	117	
M1	38	30	8	**0.049**
				
*Stage*				
IA	3	0	3	
IB	2	2	0	
IIA	59	31	28	
IIB	246	160	86	
III	0	0	0	
IV	38	30	8	**0.009** a
				
*Tumour histological grade* b				
W/D	90	41	49	
M/D	181	122	59	
P/D	77	60	17	**<0.0001** a
				
*Tumour margin status*				
Negative	249	166	82	
Positive	100	57	43	0.085
				
*Nerve plexus invasion* b				
Absence	112	65	47	
Presence	236	158	78	0.120
				
*Lymphatic invasion* b				
0, 1	102	58	44	
2, 3	246	165	81	0.086
				
*Venous invasion* b				
0, 1	124	63	61	
2, 3	224	160	64	**0.0002**
				
*Intrapancreatic neural invasion* b				
0, 1	141	86	55	
2, 3	207	137	70	0.363
				
*Recurrent site* c				
Local	54	39	15	
Distant sites	198	134	64	0.620
				
*Expression of CAIX in cancer cells* d				
Absence	114	69	45	
Presence	89	63	26	0.143
				
*Expression of CAIX in stromal cells* d				
Absence	74	31	43	
Presence	129	101	28	**<0.0001**
Total	348	223	125	

Abbreviations: CAIX=carbonic anhydrase IX; M/D=moderately differentiated adenocarcinoma; P/D=poorly differentaited adenocarcinoma; W/D=well-differentiated adenocarcinoma.

a Comparisons of qualitative variables are performed using the *χ*^2^ test, and otherwise by Fisher’s exact test.

b Classified according to the classification of pancreatic carcinoma of Japan Pancreas Society.

c Number of patients with tumour recurrence was 252.

d Number of patients used in the immunohistochemical analysis was 203. Statistically significant in bold values.

**Table 2:** Univariate and multivariate analyses of prognostic factors associated with disease-free survival in patients with ductal carcinoma of the pancreas (*n*=348)

	**Univariate analysis**		**Multivariate analysis**	
**Variables**	**HR (95% CI)**	***P*-value**	**HR (95% CI)**	***P*-value**
Age (⩾60 years/<60 years)	1.159 (0.895–1.501)	0.263		
Gender (male/female)	0.987 (0.764–1.274)	0.919		
Localisation (pancreas head/body or tail)	0.865 (0.657–1.140)	0.304		
Tumour size (⩾30 mm/<30 mm)	1.789 (1.306–2.450)	**0.0003**		
Pathologic tumour status (T1+T2/T3)	3.863 (1.235–12.1)	**0.020**		
Pathologic node status (N0/N1)	2.028 (1.416–2.906)	**0.0001**		
Pathologic metastasis status (M0/M1)	2.258 (1.550–3.289)	**<0.0001**	2.042 (1.387–3.006)	**0.0003**
Histological grade (W/D/M/D, P/D)^a^	1.507 (1.122–2.025)	**0.006**		
Tumour margin status (negative/positive)	1.470 (1.121–1.929)	**0.005**		
PL (absence/presence)^a^	1.560 (1.182–2.060)	**0.002**		
Lymphatic invasion (0, 1/2, 3)^a^	2.040 (1.513–2.751)	**<0.0001**	1.475 (1.068–2.038)	**0.018**
Venous invasion (0, 1/2, 3)^a^	1.985 (1.508–2.614)	**<0.0001**	1.474 (1.097–1.980)	**0.010**
Intrapancreatic neural invasion (0, 1/2, 3)^a^	1.655 (1.269–2.157)	**0.0002**	1.506 (1.145–1.981)	**0.003**
Histological necrosis (absence/presence)	2.007 (1.531–2.630)	**<0.0001**	1.853 (1.407–2.440)	**<0.0001**
				
Univariate and multivariate analyses of prognostic factors associated with disease-specific survival in patients with ductal carcinoma of the pancreas (n=348)				
	**Univariate analysis**		**Multivariate analysis**	
**Variables**	**HR (95% CI)**	***P*-value**	**HR (95% CI)**	***P*-value**
Age (⩾60 years/<60 years)	1.054 (0.811–1.371)	0.692		
Gender (male/female)	0.935 (0.721–1.213)	0.615		
Localisation (pancreas head/body or tail)	0.829 (0.628–1.096)	0.189		
Tumour size (⩾30 mm/<30 mm)	1.890 (1.371–2.605)	**0.0001**		
Pathologic tumour status (T1+T2/T3)	6.333 (1.572–25.5)	**0.009**		
Pathologic node status (N0/N1)	2.024 (1.406–2.915)	**0.0002**		
Pathologic metastasis status (M0/M1)	2.199 (1.509–3.204)	**<0.0001**	1.839 (1.252–2.700)	**0.002**
Histological grade (W/D/M/D, P/D)^a^	1.611 (1.193–2.176)	**0.002**		
Tumour margin status (negative/positive)	1.555 (1.183–2.043)	**0.002**	1.379 (1.038–1.833)	**0.027**
PL (absence/presence)^a^	1.690 (1.267–2.253)	**0.0004**		
Lymphatic invasion (0, 1/2, 3)^a^	2.409 (1.762–3.293)	**<0.0001**	1.992 (1.440–2.757)	**<0.0001**
Venous invasion (0, 1/2, 3)^a^	1.968 (1.486–2.607)	**<0.0001**		
Intrapancreatic neural invasion (0, 1/2, 3)^a^	1.709 (1.305–2.238)	**<0.0001**	1.443 (1.087–1.915)	**0.011**
Histological necrosis (absence/presence)	2.196 (1.659–2.905)	**<0.0001**	2.238 (1.686–2.971)	**<0.0001**

Abbreviations: CI=confidence interval; HR=hazards ratio; M/D=moderately differentiated adenocarcinoma; P/D=poorly differentaited adenocarcinoma; PL=nerve plexus invasion; W/D=well-differentiated adenocarcinoma.

a Classified according to the classification of pancreatic carcinoma of Japan Pancreas Society

## References

[bib1] Adsay NV, Basturk O, Bonnett M, Kilinc N, Andea AA, Feng J, Che M, Aulicino MR, Levi E, Cheng JD (2005) A proposal for a new and more practical grading scheme for pancreatic ductal adenocarcinoma. Am J Surg Pathol 29: 724–733 15897739 10.1097/01.pas.0000163360.40357.f1

[bib2] Bristow RG, Hill RP (2008) Hypoxia and metabolism. Hypoxia, DNA repair and genetic instability. Nat Rev Cancer 8: 180–192 18273037 10.1038/nrc2344

[bib3] Center for Cancer Control and Information Services, National Cancer Center Japan (2009) Cancer Statistics in Japan

[bib4] Chia SK, Wykoff CC, Watson PH, Han C, Leek RD, Pastorek J, Gatter KC, Ratcliffe P, Harris AL (2001) Prognostic significance of a novel hypoxia-regulated marker, carbonic anhydrase IX, in invasive breast carcinoma. J Clin Oncol 19: 3660–3668 11504747 10.1200/JCO.2001.19.16.3660

[bib5] Couvelard A, O’Toole D, Leek R, Turley H, Sauvanet A, Degott C, Ruszniewski P, Belghiti J, Harris AL, Gatter K, Pezzella F (2005) Expression of hypoxia-inducible factors is correlated with the presence of a fibrotic focus and angiogenesis in pancreatic ductal adenocarcinomas. Histopathology 46: 668–676 15910598 10.1111/j.1365-2559.2005.02160.x

[bib6] Fleiss JL (1971) Measuring nominal scale agreement among many rates. Pshycol Bull 76: 378–382

[bib7] Gilchrist KW, Gray R, Fowble B, Tormey DC, Taylor SGt (1993) Tumor necrosis is a prognostic predictor for early recurrence and death in lymph node-positive breast cancer: a 10-year follow-up study of 728 Eastern Cooperative Oncology Group patients. J Clin Oncol 11: 1929–1935 8410120 10.1200/JCO.1993.11.10.1929

[bib8] Grothey A, Galanis E (2009) Targeting angiogenesis: progress with anti-VEGF treatment with large molecules. Nat Rev Clin Oncol 6: 507–518 19636328 10.1038/nrclinonc.2009.110

[bib9] Harris AL (2002) Hypoxia – a key regulatory factor in tumour growth. Nat Rev Cancer 2: 38–47 11902584 10.1038/nrc704

[bib10] Hruban RH, Pitman MB, Klimstra DS (2007) Ductal adenocarcinoma. In AFIP Atlas of Tumor Pathology. Tumors of the Pancreas, Hruban RH, Pitman MB, Klimstra DS (eds) 4th edn, pp 111–164. ARP Press: Washington, DC

[bib11] Japan Pancreas Society (2003) Classification of Pancreatic Cancer. 2nd English edn, Kanehara: Tokyo, Japan

[bib12] Jemal A, Siegel R, Ward E, Hao Y, Xu J, Thun MJ (2009) Cancer statistics, 2009. CA Cancer J Clin 59: 225–249 19474385 10.3322/caac.20006

[bib13] Kitada T, Seki S, Sakaguchi H, Sawada T, Hirakawa K, Wakasa K (2003) Clinicopathological significance of hypoxia-inducible factor-1alpha expression in human pancreatic carcinoma. Histopathology 43: 550–555 14636255 10.1111/j.1365-2559.2003.01733.x

[bib14] Klöppel G, Adler G, Hruban RH, Kern SE, Longnecker DS, Partanen TJ (2000) Ductal adenocarcinoma of the pancreas. In World Health Organization Classification of Tumours. Pathology & Genetics. Tumours of the Digestive System, Hamilton SR, Aaltonen LA (eds), pp 221–230. IARC Press: Lyon

[bib15] Landis JR, Koch GG (1977) The measurement of observer agreement for categorical data. Biometrics 33: 159–174 843571

[bib16] Lim JE, Chien MW, Earle CC (2003) Prognostic factors following curative resection for pancreatic adenocarcinoma: a population-based, linked database analysis of 396 patients. Ann Surg 237: 74–85 12496533 10.1097/00000658-200301000-00011 PMC1513971

[bib17] Luttges J, Schemm S, Vogel I, Hedderich J, Kremer B, Klöppel G (2000) The grade of pancreatic ductal carcinoma is an independent prognostic factor and is superior to the immunohistochemical assessment of proliferation. J Pathol 191: 154–161 10861575 10.1002/(SICI)1096-9896(200006)191:2<154::AID-PATH603>3.0.CO;2-C

[bib18] Mitsunaga S, Hasebe T, Iwasaki M, Kinoshita T, Ochiai A, Shimizu N (2005) Important prognostic histological parameters for patients with invasive ductal carcinoma of the pancreas. Cancer Sci 96: 858–865 16367904 10.1111/j.1349-7006.2005.00128.x PMC11158361

[bib19] Mitsunaga S, Hasebe T, Kinoshita T, Konishi M, Takahashi S, Gotohda N, Nakagohri T, Ochiai A (2007) Detail histologic analysis of nerve plexus invasion in invasive ductal carcinoma of the pancreas and its prognostic impact. Am J Surg Pathol 31: 1636–1644 18059219 10.1097/PAS.0b013e318065bfe6

[bib20] Nakatsura T, Hasebe T, Tsubono Y, Ryu M, Kinoshita T, Kawano N, Konishi M, Kosuge T, Kanai Y, Mukai K (1997) Histological prognostic parameters for adenocarcinoma of the pancreatic head. Proposal for a scoring system for prediction of outcome. J Hep Bil Pancr Surg 4: 441–448

[bib21] Ord JJ, Agrawal S, Thamboo TP, Roberts I, Campo L, Turley H, Han C, Fawcett DW, Kulkarni RP, Cranston D, Harris AL, Ord JJ, Agrawal S, Thamboo TP, Roberts I, Campo L, Turley H, Han C, Fawcett DW, Kulkarni RP, Cranston D, Harris AL (2007) An investigation into the prognostic significance of necrosis and hypoxia in high grade and invasive bladder cancer. J Urol 178: 677–682 17574616 10.1016/j.juro.2007.03.112

[bib22] Pastorekova S, Zavadova Z, Kostal M, Babusikova O, Zavada J (1992) A novel quasi-viral agent, MaTu, is a two-component system. Virology 187: 620–626 1312272 10.1016/0042-6822(92)90464-z

[bib23] Philip PA, Mooney M, Jaffe D, Eckhardt G, Moore M, Meropol N, Emens L, O’Reilly E, Korc M, Ellis L, Benedetti J, Rothenberg M, Willett C, Tempero M, Lowy A, Abbruzzese J, Simeone D, Hingorani S, Berlin J, Tepper J (2009) Consensus report of the national cancer institute clinical trials planning meeting on pancreas cancer treatment. J Clin Oncol 27: 5660–5669 19858397 10.1200/JCO.2009.21.9022 PMC7587401

[bib24] Schnelldorfer T, Ware AL, Sarr MG, Smyrk TC, Zhang L, Qin R, Gullerud RE, Donohue JH, Nagorney DM, Farnell MB (2008) Long-term survival after pancreatoduodenectomy for pancreatic adenocarcinoma: is cure possible? Ann Surg 247: 456–462 18376190 10.1097/SLA.0b013e3181613142

[bib25] Semenza GL (2006) Development of novel therapeutic strategies that target HIF-1. Expert Opin Ther Targets 10: 267–280 16548775 10.1517/14728222.10.2.267

[bib26] Semenza GL (2009) HIF-1 inhibitors for cancer therapy: from gene expression to drug discovery. Curr Pharm Des 15: 3839–3843 19671047 10.2174/138161209789649402

[bib27] Sessa C, Guibal A, Del Conte G, Ruegg C (2008) Biomarkers of angiogenesis for the development of antiangiogenic therapies in oncology: tools or decorations? Nat Clin Pract Oncol 5: 378–391 18560389 10.1038/ncponc1150

[bib28] Shimada K, Sakamoto Y, Sano T, Kosuge T, Hiraoka N (2006) Reappraisal of the clinical significance of tumor size in patients with pancreatic ductal carcinoma. Pancreas 33: 233–239 17003643 10.1097/01.mpa.0000232917.78890.01

[bib29] Sohn TA, Yeo CJ, Cameron JL, Koniaris L, Kaushal S, Abrams RA, Sauter PK, Coleman J, Hruban RH, Lillemoe KD (2000) Resected adenocarcinoma of the pancreas-616 patients: results, outcomes, and prognostic indicators. J Gastrointest Surg 4: 567–579 11307091 10.1016/s1091-255x(00)80105-5

[bib30] Sun HC, Qiu ZJ, Liu J, Sun J, Jiang T, Huang KJ, Yao M, Huang C, Sun H-C, Qiu Z-J, Liu J, Sun J, Jiang T, Huang K-J, Yao M, Huang C (2007) Expression of hypoxia-inducible factor-1 alpha and associated proteins in pancreatic ductal adenocarcinoma and their impact on prognosis. Int J Oncol 30: 1359–1367 17487356

[bib31] Takahashi Y, Akishima-Fukasawa Y, Kobayashi N, Sano T, Kosuge T, Nimura Y, Kanai Y, Hiraoka N (2007) Prognostic value of tumor architecture, tumor-associated vascular characteristics, and expression of angiogenic molecules in pancreatic endocrine tumors. Clin Cancer Res 13: 187–196 17200354 10.1158/1078-0432.CCR-06-1408

[bib32] Takai S, Satoi S, Toyokawa H, Yanagimoto H, Sugimoto N, Tsuji K, Araki H, Matsui Y, Imamura A, Kwon AH, Kamiyama Y (2003) Clinicopathologic evaluation after resection for ductal adenocarcinoma of the pancreas: a retrospective, single-institution experience. Pancreas 26: 243–249 12657950 10.1097/00006676-200304000-00007

[bib33] Trede M, Schwall G, Saeger HD (1990) Survival after pancreatoduodenectomy. 118 consecutive resections without an operative mortality. Ann Surg 211: 447–458 2322039 10.1097/00000658-199004000-00011 PMC1358031

[bib34] Vaupel P, Mayer A (2007) Hypoxia in cancer: significance and impact on clinical outcome. Cancer Metastasis Rev 26: 225–239 17440684 10.1007/s10555-007-9055-1

[bib35] Wittekind C, Greene FL, Hutter RVP, Klimpfinger M, Sobin LH (2005) UICC TNM Atlas 5th edn, Springer: New York, NY

[bib36] Yeo CJ, Cameron JL, Lillemoe KD, Sitzmann JV, Hruban RH, Goodman SN, Dooley WC, Coleman J, Pitt HA (1995) Pancreaticoduodenectomy for cancer of the head of the pancreas. 201 patients. Ann Surg 221: 721–731; discussion 731–733 7794076 10.1097/00000658-199506000-00011 PMC1234702

[bib37] Zavada J, Zavadova Z, Pastorek J, Biesova Z, Jezek J, Velek J (2000) Human tumour-associated cell adhesion protein MN/CA IX: identification of M75 epitope and of the region mediating cell adhesion. Br J Cancer 82: 1808–1813 10839295 10.1054/bjoc.2000.1111 PMC2363230

