# Supplementary material for: Tumour necrosis is a postoperative prognostic marker for pancreatic cancer patients with a high interobserver reproducibility in histological evaluation
Source: Br J Cancer. 2010 Aug 24;103(7):1057–65. doi: 10.1038/sj.bjc.6605854 (PMC2965866; doi:10.1038/sj.bjc.6605854)
Supplement: Supplementary file 1 [file 6605854x1.doc]

Supplementary Table 1. Univariate and multivariate analyses of prognostic factors associated with disease-free survival in patients with ductal carcinoma of the pancreas (n=203)

Variables	Univariate analysis HR (95% CI)	*P* value	Multivariate analysis HR (95% CI)	*P* value
Age (60 years/ < 60 years)	1.051 (0.450-1.473)	0.772		
Gender (male/ female)	0.952 (0.683-1.326)	0.770		
Localization (pancreas head/ body or tail)	0.976 (0.691-1.378)	0.889		
Tumor size (30 mm/ <30 mm)	1.880 (1.257-2.812)	**0.002**		
Pathologic tumor status (T1+T2/ T3)	5.029 (1.702-36.0)	0.108		
Pathologic node status (N0/ N1)	1.965 (1.224-3.153)	**0.005**		
Pathologic metastasis status (M0/ M1)	2.552 (1.587-4.103)	**0.0001**	2.896 (1.781-4.708)	**<0.0001**
Histological grade (W/D/ M/D, P/D)*	1.906 (1.275-2.848)	**0.002**		
Tumor margin status (negative/ positive)	1.231 (0.853-1.778)	0.267		
PL (absence/ presence)*	1.415 (0.995-2.012)	0.053		
Lymphatic invasion (0, 1/ 2, 3)*	1.930 (1.308-2.848)	**0.0009**		
Venous invasion (0, 1/ 2, 3)*	2.003 (1.412-2.841)	**< 0.0001**	1.919 (1.350-2.728)	**0.0003**
Intrapancreatic neural invasion (0, 1/ 2, 3)*	1.545 (1.104-2.162)	**0.011**		
Expression of CAIX in cancer cells (absence/ presence)	1.437 (1.038-1.990)	**0.028**		
Expression of CAIX in stromal cells (absence/ presence)	1.647 (1.171-2.318)	**0.004**	1.647 (1.164-2.331)	**0.005**

Univariate and multivariate analyses of prognostic factors associated with disease-specific survival in patients with ductal carcinoma of the pancreas (n=203)

Variables	Univariate analysis HR (95% CI)	*P* value	Multivariate analysis HR (95% CI)	*P* value
Age (60 years/ < 60 years)	1.056 (0.746-1.493)	0.760		
Gender (male/ female)	0.838 (0.591-1.187)	0.320		
Localization (pancreas head/ body or tail)	0.902 (0.631-1.288)	0.569		
Tumor size (30 mm/ <30 mm)	1.829 (1.206-2.776)	**0.005**		
Pathologic tumor status (T1+T2/ T3)	5.524 (0.772-39.6)	0.089		
Pathologic node status (N0/ N1)	1.966 (1.209-3.198)	**0.006**		
Pathologic metastasis status (M0/ M1)	2.524 (1.548-4.115)	**0.0002**	2.289 (1.390-3.769)	**0.001**
Histological grade (W/D/ M/D, P/D)*	2.043 (1.331-3.136)	**0.001**	1.638 (1.048-2.560)	**0.03**
Tumor margin status (negative/ positive)	1.472 (1.011-2.143)	**0.044**		
PL (absence/ presence)*	1.457 (1.010-2.101)	**0.044**		
Lymphatic invasion (0, 1/ 2, 3)*	2.448 (1.603-3.738)	**<0.0001**	2.138 (1.390-3.289)	**0.0005**
Venous invasion (0, 1/ 2, 3)*	1.849 (1.290-2.651)	**0.0008**		
Intrapancreatic neural invasion (0, 1/ 2, 3)*	1.610 (1.136-2.283)	**0.007**		
Expression of CAIX in cancer cells (absence/ presence)	1.384 (0.989-1.938)	0.057		
Expression of CAIX in stromal cells (absence/ presence)	1.730 (1.208-2.478)	**0.003**	1.622 (1.116-2.356)	**0.011**

W/D, well differentiated adenocarcinoma; M/D, moderately differentiated adenocarcinoma; P/D, poorly differentaited adenocarcinoma; PL, nerve plexus invasion

*Classified according to the classification of pancreatic carcinoma of Japan Pancreas Society

Supplementary Table 2. Univariate and multivariate analyses of prognostic factors associated with disease-free survival in patients with ductal carcinoma of the pancreas (n=348)

	Univariate analysis		Multivariate analysis	
Variables	HR (95% CI)	*P* value	HR (95% CI)	*P* value
Age (60 years/ < 60 years)	1.159 (0.895-1.501)	0.263		
Gender (male/ female)	0.987 (0.764-1.274)	0.919		
Localization (pancreas head/ body or tail)	0.865 (0.657-1.140)	0.304		
Tumor size (30 mm/ <30 mm)	1.789 (1.306-2.450)	**0.0003**		
Pathologic tumor status (T1+T2/ T3)	3.863 (1.235-12.1)	**0.020**		
Pathologic node status (N0/ N1)	2.028 (1.416-2.906)	**0.0001**		
Pathologic metastasis status (M0/ M1)	2.258 (1.550-3.289)	**<0.0001**	2.100 (1.424-3.097)	**0.0002**
Histological grade (W/D/ M/D, P/D)*	1.507 (1.122-2.025)	**0.006**		
Tumor margin status (negative/ positive)	1.470 (1.121-1.929)	**0.005**		
PL (absence/ presence)*	1.560 (1.182-2.060)	**0.002**		
Lymphatic invasion (0, 1/ 2, 3)*	2.040 (1.513-2.751)	**<0.0001**	1.497 (1.087-2.063)	**0.014**
Venous invasion (0, 1/ 2, 3)*	1.985 (1.508-2.614)	**< 0.0001**	1.426 (1.057-1.923)	**0.020**
Intrapancreatic neural invasion (0, 1/ 2, 3)*	1.655 (1.269-2.157)	**0.0002**	1.539 (1.170-2.025)	**0.002**
Massive necrosis (absence/ presence)	1.767 (1.340-2.329)	**<0.0001**	1.661 (1.241-2.223)	**0.0006**
Micronecrosis (absence/ presence)	1.444 (1.121-1.859)	**0.004**	1.604 (1.233-2.085)	**0.0004**

Univariate and multivariate analyses of prognostic factors associated with disease-specific survival in patients with ductal carcinoma of the pancreas (n=348)

	Univariate analysis		Multivariate analysis	
Variables	HR (95% CI)	*P* value	HR (95% CI)	*P* value
Age (60 years/ < 60 years)	1.054 (0.811-1.371)	0.692		
Gender (male/ female)	0.935 (0.721-1.213)	0.615		
Localization (pancreas head/ body or tail)	0.829 (0.628-1.096)	0.189		
Tumor size (30 mm/ <30 mm)	1.890 (1.371-2.605)	**0.0001**		
Pathologic tumor status (T1+T2/ T3)	6.333 (1.572-25.5)	**0.009**		
Pathologic node status (N0/ N1)	2.024 (1.406-2.915)	**0.0002**		
Pathologic metastasis status (M0/ M1)	2.199 (1.509-3.204)	**<0.0001**	1.954 (1.330-2.869)	**0.0006**
Histological grade (W/D/ M/D, P/D)*	1.611 (1.193-2.176)	**0.002**		
Tumor margin status (negative/ positive)	1.555 (1.183-2.043)	**0.002**	1.396 (1.048-1.859)	**0.023**
PL (absence/ presence)*	1.690 (1.267-2.253)	**0.0004**		
Lymphatic invasion (0, 1/ 2, 3)*	2.409 (1.762-3.293)	**<0.0001**	2.011 (1.486-2.779)	**<0.0001**
Venous invasion (0, 1/ 2, 3)*	1.968 (1.486-2.607)	**< 0.0001**		
Intrapancreatic neural invasion (0, 1/ 2, 3)*	1.709 (1.305-2.238)	**<0.0001**	1.449 (1.093-1.921)	**0.010**
Massive necrosis (absence/ presence)	1.841 (1.390-2.438)	**< 0.0001**	2.069 (1.544-2.771)	**<0.0001**
Micronecrosis (absence/ presence)	1.535 (1.188-1.984)	**0.001**	1.823 (1.398-2.377)	**<0.0001**

W/D, well differentiated adenocarcinoma; M/D, moderately differentiated adenocarcinoma; P/D, poorly differentaited adenocarcinoma; PL, nerve plexus invasion

*Classified according to the classification of pancreatic carcinoma of Japan Pancreas Society
